# Evolution of Topological Surface States Following Sb Layer Adsorption on Bi_2_Se_3_

**DOI:** 10.3390/ma14071763

**Published:** 2021-04-02

**Authors:** Kris Holtgrewe, Conor Hogan, Simone Sanna

**Affiliations:** 1 Institut für Theoretische Physik, Justus-Liebig-Universität Gießen, 35392 Gießen, Germany; simone.sanna@theo.physik.uni-giessen.de; 2 Center for Materials Research (LaMa), Justus-Liebig-Universität Gießen, 35392 Gießen, Germany; 3 Istituto di Struttura della Materia, Consiglio Nazionale delle Ricerche (ISM-CNR), Via del Fosso del Cavaliere 100, 00133 Roma, Italy; Conor.Hogan@ism.cnr.it

**Keywords:** surface science, topological insulator, topological surface states, topological phase transition, van der waals, hetero structure, bismuth selenide, adsorption, density functional theory, ab initio

## Abstract

Thin antimony layers adsorbed on bismuth selenide (${\mathrm{Bi}}_{2}{\mathrm{Se}}_{3}$) present an exciting topological insulator system. Much recent effort has been made to understand the synthesis and electronic properties of the heterostructure, particularly the migration of the topological surface states under adsorption. However, the intertwinement of the topological surface states of the pristine ${\mathrm{Bi}}_{2}{\mathrm{Se}}_{3}$ substrate with the Sb adlayer remains unclear. In this theoretical work, we apply density functional theory (DFT) to model heterostructures of single and double atomic layers of Sb on a bismuth selenide substrate. We thereby discuss established and alternative structural models, as well as the hybridization of topological surface states with the Sb states. Concerning the geometry, we reveal the possibility of structures with inverted Sb layers which are energetically close to the established ones. The formation energy differences are below 10 meV/atom. Concerning the hybridization, we trace the band structure evolution as a function of the adlayer-substrate distance. By following changes in the connection between the Kramers pairs, we extract a series of topological phase transitions. This allows us to explain the origin of the complex band structure, and ultimately complete our knowledge about this peculiar system.

## 1. Introduction

*Topological insulators* (TIs) have attracted huge interest from the condensed matter research community in the past decade [1,2,3,4,5,6,7,8,9,10,11,12,13]. Their bulk band structures feature a band gap, like those of ordinary band insulators (conventional insulator, CI), but they differ from the latter in some topological invariants. Consequently, a continuous, gap-preserving transformation between the respective valence and conduction bands of the TI and the CI does not exist. So when a TI makes contact with a CI, a gap closing has to occur that is accompanied by metallic edge states somewhere at the interface. These edge states are *topologically protected*, so they are indestructible by any perturbation which does not change the topological characters of the TI and the CI. In particular, edge states form at the interface between a TI and vacuum which are called topological surface states (TSSs).

Bismuth selenide (${\mathrm{Bi}}_{2}{\mathrm{Se}}_{3}$, BS) is a $Z_{2}$ insulator that gets its topological character from time reversal symmetry along with a strong spin-orbit coupling (SOC), leading to an inversion of the bands at the Γ point [14]. When cut along the (0001) plane of the hexagonal crystal, the TSSs show a Γ-centered, cone-like dispersion with zero spin expectation value parallel to the surface wave vector $k_{\|}$ [15]. The Fermi surface is a single circle whose opposite points are Kramers degenerate. Hence, elastic backscattering at time-reversal-symmetry preserving defects is forbidden and the metallicity is insensitive to disorder. The 2D surface cones are called *Dirac cones*, as they are analogous to the helical solutions of the massless and free Dirac equation. So, the BS surface presents an exciting opportunity to realize and explore models for such theoretical constructs.

A further step towards advanced functionality is the tuning and manipulation of the TSS. As BS is a van der Waals (vdW) material consisting of weakly bound quintuple layers (QLs), the unreconstructed (0001) surface has no unsaturated bonds, suggesting that layers will bind to it by means of vdW interactions. Antimony-based adsorbates have proven to be quite interesting because antimony (Sb) appears in different allotropes which also have a layered crystal structure [16,17]. As the β phase of antimony (β-Sb, grey antimony) consists of hexagonal bilayers (BLs) which have a lattice constant similar to that of the BS surface, a lattice matched adsorption of single BLs on BS is possible. The growth of 1BL and 2BL of β-Sb on BS samples (1BL-Sb@BS and 2BL-Sb@BS) has been realized [18,19] and controlled by a temperature-induced phase transition [20,21].

The impact of the Sb adsorption on the electronic band structure of the BS substrate is complex. Strong SOC makes bulk β-Sb a $Z_{2}$ topological semimetal, while few BL thick slabs are topologically trivial [22]. The interplay between the Sb bands and the TSS has been investigated in several works [19,23]. Under Sb adsorption, the TSS migrates from the top BS QL to the top Sb BL, while the spin texture of the Dirac cone is preserved. The CI can, thus, be thought as being “topologized” under presence of the topological BS substrate. As a consequence, the TI/CI interface is not between BS substrate and the Sb layer, but at the surface of the Sb layer [24].

This work provides new insight into the structural and electronic properties of the Sb@BS system. So far, the structure of 1BL-Sb@BS has been derived from a continued stacking of the honeycomb-like Sb BL on the buckled, honeycomb-like surface of the BS. However, the vdW coupling at the hetero-junction makes other stackings possible, e.g., a lateral shift of the BL or an inversion of the buckled honeycomb. In addition, for the second BL, structures different from a continued bulk stacking cannot be excluded a priori. Hence, the first part of this work contains a careful analysis of these alternative structural models and their impact on the electronic band structure. The second part deals with the hybridization between the substrate and the adlayer, and highlights the reordering of the bands as a function of the BS-Sb distance. This provides a better understanding of the origin of the features in the final electronic band structures and of the topological mechanism.

## 2. Results

### 2.1. Structure of Bulk ${\mathrm{Bi}}_{2}{\mathrm{Se}}_{3}$

Bulk ${\mathrm{Bi}}_{2}{\mathrm{Se}}_{3}$ (BS) is a layered material consisting of covalently bound sheets which are in turn more loosely bound to each other. One sheet consists of five primitively hexagonal layers which stack as a Se-Bi-Se-Bi-Se quintuple layer (QL) in an “(ABCAB)” stacking order. As the next QL starts with a Se atom at the “C” site, the vertical periodicity is 3QL.

The structural optimization of bulk BS returns an equilibrium (hexagonal) cell volume of (425.25±0.01) Å^3^. The cell parameters are $a_{\mathrm{BS}}=4.13$ Å and $c_{\mathrm{BS}}=28.73$ Å and the Wyckoff position parameters for the trigonal cell are u=0.4000 and v=0.2111. This translates to an inter-layer distance of $d_{\mathrm{BS}}=2.56$ Å and a layer thickness of $w_{\mathrm{BS}}=7.02$ Å. The theoretical results reproduce well experimental findings with deviations below 1% (see Table 1). Test calculations show that the inclusion of SOC has a moderate influence (∼5%), and the vdW treatment has a strong influence (∼5%) on the structural parameters, especially on the inter-layer distance. The moderate influence of SOC on the equilibrium geometry was also observed for other layered materials, e.g., transition metal dichalcogenide compounds [25].

### 2.2. Structure of Bulk Sb and Isolated Sb Sheets

Like bismuth selenide, β-Sb is a layered material, with the difference that the sheets are composed of buckled honeycomb Sb, or equivalently, bilayers (BLs) of two hexagonal atomic layers. Like in BS, the stacking order is “(AB)(CA)(BC)”, which leads to a vertical 3BL periodicity. Beside the natural bulk β-Sb, further modified β-Sb structures are investigated. The first variation for the bulk is an inversion of every second BL and an adapted stacking continuation, which results in an “(AB)(CB)” order with a 2BL periodicity. The second bulk variation is a distorted structure, where the stacking order is “(AB)” with a 1BL periodicity. In addition, the energies of a natural bulk cell matched to the lattice parameters of the BS surface ($a=a_{\mathrm{BS}}$) and of free standing sheets of β-Sb (1BL, 2BL, and 2BL with inverted stacking) are calculated. For stability considerations, the chemical potential of the Sb atoms $\mu_{\mathrm{Sb}}$ for each structure is determined by division of the DFT total energies by the number of Sb atoms $N_{\mathrm{Sb}}$. They are referred to that of natural bulk β-Sb, so they can be interpreted as formation energies. The results may be found along with the structural parameters (lateral lattice constant $a_{\mathrm{Sb}}$ and the inter-layer distance $d_{\mathrm{Sb}}$) in Table 2. The values for the natural bulk phase and the free standing sheet are consistent with Reference [17].

### 2.3. Structural Details of the Sb@BS System

The BS surface is modeled with a slab derived from the optimized bulk cell. The difference from the one in Reference [24] is, first, that it uses lattice and internal parameters from the DFT calculation, while the latter has been built from experimental data, and, second, that the whole top QL is free to relax during surface relaxation. This has a minor effect on the thickness of the top QL $w_{\mathrm{BS}}^{\mathrm{top}}=7.02$ Å, while the inter-layer distance between the top QL and the second top QL increases slightly to $d_{\mathrm{BS}}^{\mathrm{top}}=2.57$ Å ($\Delta d_{\mathrm{BS}}=+0.01$Å). The smallness of the movements in the top QL indicates that the unfixing of further QLs in the slab has no physically relevant influence.

**Figure 1:**
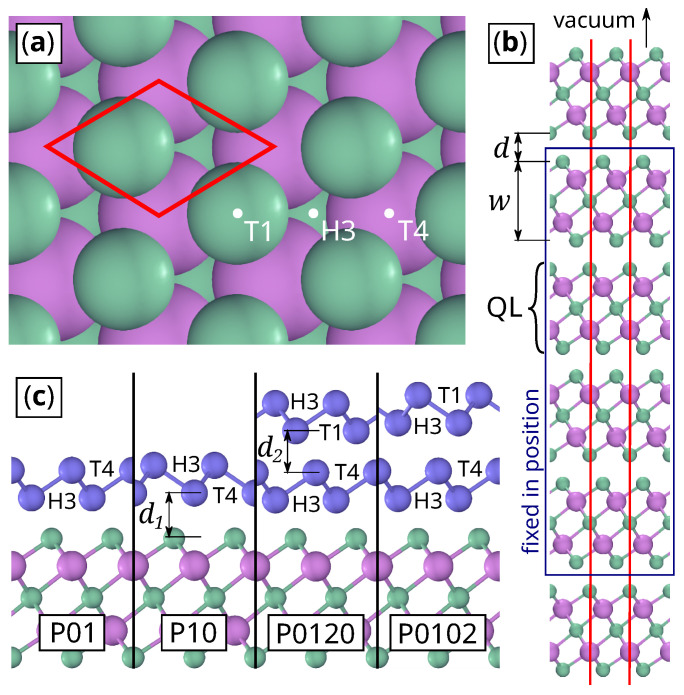
Sketch of the Bi_2_S_3_ slab. Bi atoms are violet, Se atoms are green, Sb atoms are blue: (**a**) Top view with unit cell (red) and labels for symmetry positions (white). (**b**) Cross section with unit cell (red). The atoms in the blue box are fixed in position during relaxation. (**c**) Cross section of the two most stable 1BL-Sb@BS and 2BL-Sb@BS structures.

The pristine BS surface has three-fold rotational symmetry and contains three high-symmetry points: T1 occupied by the top Se atom, T4 occupied by the top Bi atom, and H3 occupied by the second top Se atom (Figure 1a). When β-Sb sheets adsorb to the BS surface, one might expect the same stacking as in the bulk, i.e., with the next atom at H3 (Figure 1b). Thus, the first BL would occupy the H3 and the T4 site, and the second BL the T1 and the H3 site. These are the structures studied in the literature so far [19,20,23,24].

Alternative structure models result from swapping and shifting the Sb sites. In order to systemize the search, the sites [H3,T4,T1] are mapped onto integers [0,1,2] and the structures are named “P” + the stacking of the Sb atoms, e.g., 1BL-Sb@BS (2BL-Sb@BS) with the natural stacking order is called P01 (P0120). P10 denotes 1BL-Sb@BS with inverted BL, and P0102 is 2BL-Sb@BS with inverted top BL. Two inter-layer distances characterize the structures: the vertical distance between the BS substrate and the first Sb BL $d_{1}$ and the vertical distance between the first BL and the second BL $d_{2}$ (Figure 1c).

Permutations of the Sb sites result in a set of structures which have to be compared. For this purpose, the corresponding supercells are constructed and relaxed, and the free energy per Sb atom, which equals the Sb chemical potential, is calculated:

(1) $$\mu_{\mathrm{Sb}}=\frac{1}{N_{\mathrm{Sb}}}\cdot \left(F_{\mathrm{tot}}-F_{\mathrm{BS}}\right)-\mu_{\mathrm{Sb}}^{\mathrm{b-nat}}\approx \frac{1}{N_{\mathrm{Sb}}}\cdot \left(E_{\mathrm{tot}}^{\mathrm{DFT}}-E_{\mathrm{BS}}^{\mathrm{DFT}}\right)-\mu_{\mathrm{Sb}}^{\mathrm{b-nat}}.$$

**Figure 2:**
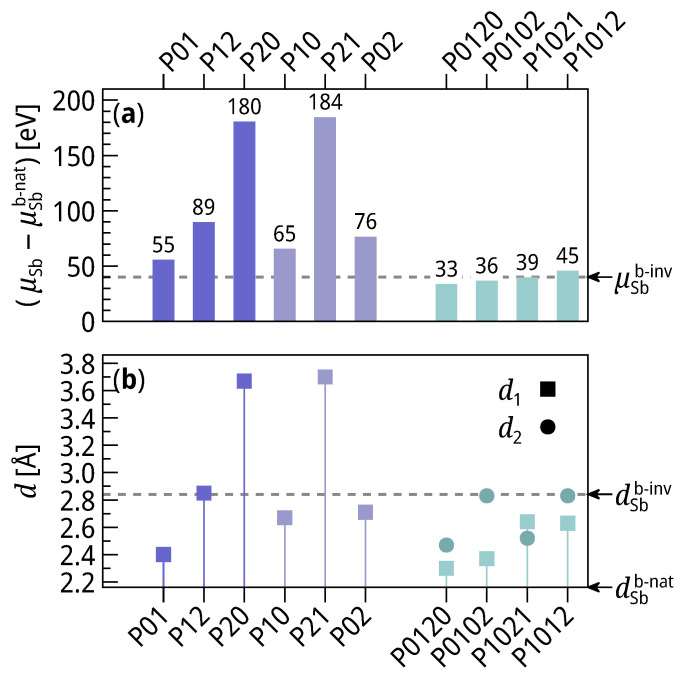
(**a**) Sb chemical potentials $\mu_{\mathrm{Sb}}$ in different structures. (**b**) Corresponding inter-layer distances $d_{\mathrm{Sb}}$ of the Sb BLs. Blue: 1BL-Sb@BS, green: 2BL-Sb@BS. Squares: distance $d_{1}$ between BS and 1st BL; circles: distance $d_{2}$ between 1st BL and 2nd BL. The vertical lines under the points for the 1st BL highlight the correlation between $d_{1}$ and $\mu_{\mathrm{Sb}}$.

$N_{\mathrm{Sb}}$ denotes the number of Sb atoms in the system, $F_{\mathrm{tot}}$ the free energy of the supercell, and $F_{\mathrm{BS}}$ the free energy of the pristine BS surface. In Equation (1), the free energies are approximated by the DFT total energies, as vibrational contributions to the entropy are expected to be of similar magnitude for all considered structures [27]. All chemical potentials $\mu_{\mathrm{Sb}}$ are referred to that of Sb in the bulk $\mu_{\mathrm{Sb}}^{\mathrm{b-nat}}$. They are, therefore, equal to the free enthalpy per Sb atom of the adsorption reaction: $\mathrm{Sb}\left(\mathrm{bulk}\right)+{\mathrm{Bi}}_{2}{\mathrm{Se}}_{3}\left(\mathrm{substrate}\right)\to \mathrm{Sb}@\mathrm{BS}$. When another Sb reactant (e.g., a free-standing sheet) is considered, the reaction enthalpy is the difference $\left(\mu_{\mathrm{Sb}}-\mu_{\mathrm{Sb}}^{\mathrm{reactant}}\right)$. A detailed discussion about the adsorption mechanism can be found in ref. [18,20]. The chemical potentials are reported in Figure 2a, and the inter-layer distances $d_{1}$ and $d_{2}$ in Figure 2b.

The set for 1BL-Sb@BS comprises 6 permutations in total, three even ones which are only a shift, and three odd ones with an inversion of the BL. P01, the structure with natural stacking order, has the lowest Sb chemical potential $\mu_{\mathrm{Sb}}^{01}=55\;\mathrm{meV}$ and is, hence, the most stable out of the six. The inverted structure P10 is the second most stable with $\mu_{\mathrm{Sb}}^{10}=65\;\mathrm{meV}$, a difference of 10 meV to P01. The two structures with the top Sb atom on the T1 site (above the top Se atom), i.e., P02 and P12, are the the third and fourth stable with $\mu_{\mathrm{Sb}}^{02}=76$ meV and $\mu_{\mathrm{Sb}}^{12}=89$ meV. The structures with the lower Sb atom at T1 (P20 and P21) have a high penalty on the chemical potentials with $\mu_{\mathrm{Sb}}^{20}=180\;\mathrm{meV}$ and $\mu_{\mathrm{Sb}}^{21}=184\;\mathrm{meV}$. A comparison of the inter-layer distances $d_{1}$ with the Sb chemical potentials $\mu_{\mathrm{Sb}}$ shows that these two quantities are correlated. So, the increase in distance between the BL and the BS surface reduces the binding energy.

Out of the 36 permutations which compose the 2BL-Sb@BS set, only those four samples are investigated which are expected to be the most stable ones from the results for 1BL-Sb@BS, i.e., which are derived from the natural stacking order only by BL inversions. These are the natural stacking order P0120, and the ones with simple inversions and otherwise continuous stacking order P0102, P1021 and P1012. Like in the 1BL-Sb@BS case, the most stable structure is the natural stacking P0120 with $\mu_{\mathrm{Sb}}^{0120}=33$ meV. The three other structures, however, have similar values ($\mu_{\mathrm{Sb}}^{0102}=36$ meV, $\mu_{\mathrm{Sb}}^{1021}=39$ meV, $\mu_{\mathrm{Sb}}^{1012}=45$ meV). $\mu_{\mathrm{Sb}}$ correlates with the first inter-layer distance $d_{1}$, while it seems to be independent from $d_{2}$.

### 2.4. Band Structures

**Figure 3:**
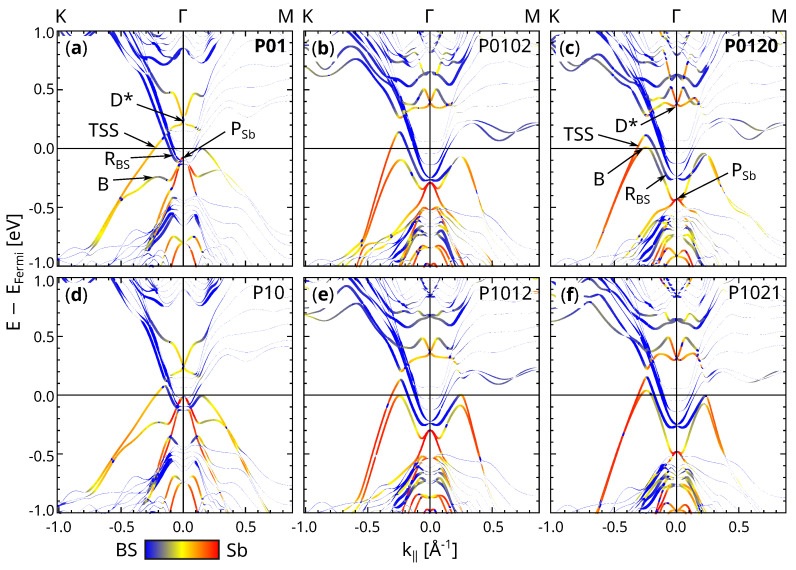
Electronic band structures for the most stable 1BL-Sb@BS and 2BL-Sb@BS structures. The band thickness corresponds to the electronic localization in the upper half of the slab. Bands localized in the Sb (BS) atoms are red (blue). (**a**–**f**) are the bands for P01, P0102, P0120, P10, P1012, P1021, respectively. Natural stacking order structures are labeled in bold font.

Figure 3 shows the band structures of the most stable models, i.e., the ones with unshifted BL inversion. The line width is proportional to the sum of the band’s PAW projections on the upper half of the slab in order to filter the surface states. The color code gives information about the localization of the band (red: Sb atoms, blue: Bi/Se atoms). The plotting scheme is the same as in Reference [24].

The band structures for the natural stacking structures (Figure 3a,c) basically reproduce the ones in Reference [24], so the slightly different approach (self-consistent cell parameters for the BS substrate and unfixing the whole top QL) has no major impact on the bands. There are five main features which characterize the band structure:

- The topological surface states “TSSs” which form at the interface between the TI and the CI.
- The Dirac point “D*”, which is the crossing of the two TSSs.
- The peak “$P_{\mathrm{Sb}}$” which is derived from in the Sb atoms.
- The band “B” which “connects” the TSS with the Sb peak.
- The bands “$R_{\mathrm{BS}}$” which stem from a Rashba splitting of the BS conduction bands.

The band structures of the two 1BL-Sb@BS structures look very similar (Figure 3a,d). D* lies above the BS conduction bands at the Γ-point. From D*, one TSS emerges upwards and one downwards, so together they form a cone-like structure. The lowest bulk BS conduction bands $R_{\mathrm{BS}}$ are split in a Rashba-like manner. Notably, the TSS and the $R_{\mathrm{BS}}$ bands, which have to cross on their way to a point of time reversal invariant momentum (TRIM), interact depending on the k-path direction. In the Γ-K direction, the crossing has no visible effect on the TSS and $R_{\mathrm{BS}}$ within the limits of the finite k-path sampling. On the other hand, a hybridization along with the formation of a clear gap occurs in the Γ-M direction. The Sb peak $P_{\mathrm{Sb}}$ touches the BS conduction bands and is the starting point for two bands. One band goes parabolically into the bulk BS valence bands, and the other forms also a hole parabola near Γ, but bends upwards for growing $k_{\|}$ and tends to the TSS after forming a local maximum. This behavior looks like a lying “B”, especially when mirrored at the k=0 axis, like in angle-resolved photo-electron spectroscopy (ARPES) experiments; therefore, it is called the “B” band. The inversion of the BL has two impacts: a lifting of $P_{\mathrm{Sb}}$ while D* drops and the opening of a small gap at the crossing of the TSS and $R_{\mathrm{BS}}$.

At first glance, the band structures of 2BL-Sb@BS look very different from those of 1BL-Sb@BS, but all the labeled features from above are still present, just changed in position and form. The adsorption of a second inverted BL on 1BL-Sb@BS (P0102 and P1012, Figure 3b,e) pushes D* and the B band upwards and $P_{\mathrm{Sb}}$ downwards. The impact is even stronger when the second BL continues the stacking of the first BL, which results in smaller $d_{2}$ (P0120 and P1021, Figure 3c,f). In addition, D* distorts from a “X”-like crossing to a Rashba-like “W” and $P_{\mathrm{Sb}}$ sinks into the BS valence bands, so that it is barely visible. In all four 2BL-Sb@BS band structures, a gap opens at the crossing of the TSS and $R_{\mathrm{BS}}$ in the Γ-K direction, in addition to the even more pronounced gap in the Γ-M direction. The gap is larger when the first BL continues the stacking of the BS (P0102 and P0120, Figure 3b,c). The largest gap forms in the natural stacking structure P0120 with the consequence that the down-bent TSS looks less steep than in the three structures with BL inversion. In all four 2BL-Sb@BS structures, the $R_{\mathrm{BS}}$ bands split more clearly, and the TSS and D* are more localized in the Sb BLs than in 1BL-Sb@BS.

### 2.5. Band Hybridization

The comparison between the band structures of 1BL-Sb@BS (Figure 3a) and 2BL-Sb@BS (Figure 3c) suggests that the addition of a second BL is a continuous transformation of the existing 1BL-Sb@BS bands without further topological phase transitions. In order to verify this, the bands are traced during a simulated peel-off and readsorption process. This enables the detection of possible band inversions, rehybridizations, and the point at which the Sb adlayer topologizes. The latter is characterized by a gap closing of the BL’s valence and conduction bands during an otherwise continuous path between start and end state.

The two inter-layer distances $d_{1}$ and $d_{2}$ (Figure 1) act as a two-dimensional reaction coordinate, which parametrizes a closed circle: First, the top BL is lifted (increasing of $d_{2}$), then the lower BL (increasing of $d_{1}$ and decreasing of $d_{2}$), and in the end, both BL adsorb to the BS surface again (decreasing of $d_{1}$), restoring the start point. The reaction coordinate is discretized into 56 samples, for which each the band structure was calculated as a snapshot.

**Figure 4:**
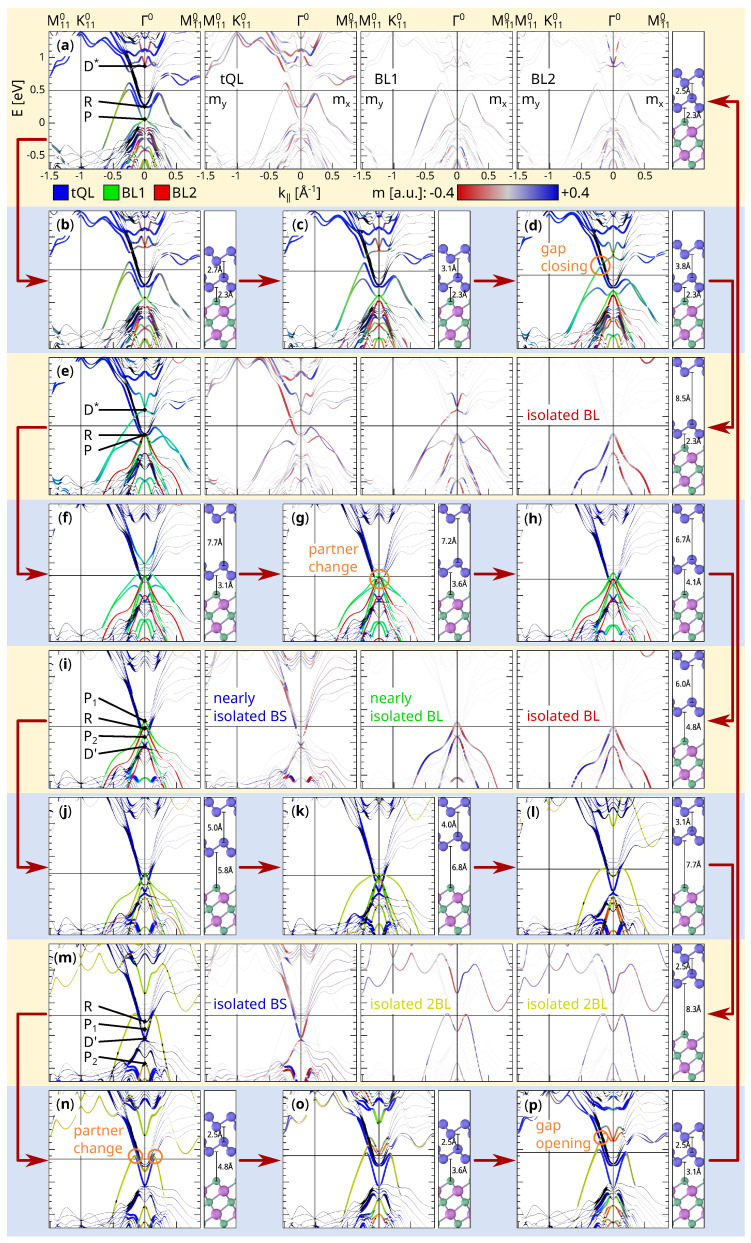
Band structure snap shots (**a**–**p**) of the closed BL peel-off and readsorption path (indicated by red arrows). First panel: Band overview (surface localization → thickness, top BS quintuple layer (tQL) → red, BL1 → green, BL2 → blue). Middle panels (**a**,**e**,**i**,**m**): Magnetization densities for tQL, BL1 and BL2, respectively (localization in respective group → thickness, negative density → blue, positive density → red). Last panel: Structure with $d_{1}$ and $d_{2}$ (reaction coordinate). Black arrows and orange marks indicate points of interest.

Figure 4 shows some of the snapshots. A complete set of images can be found in the Supplementary Material, as Supplementary Video S1. Each snapshot (row with yellow background) consists of five panels. At first, an overview of the band structure is given (first panel). Like in Figure 3, the line width corresponds to the band localization in the upper half-slab in order to filter the surface states. The color code is different, i.e., the sum of the PAW projections on the top BS quintuple layer (tQL), the lower Sb bilayer (BL1) and the upper Sb bilayer (BL2) determines the blue, green and red color channel respectively. Hence, blue bands are localized in tQL, cyan bands partially in tQL and BL1, yellow bands partially in BL1 and BL2, and black bands somewhere below tQL. The second, third, and fourth panel (not shown for snapshots in the even rows with blue background) contain the magnetization densities (projected‚ magnetization; for details, see: https://www.vasp.at/wiki/index.php/PROCAR accesss on (2 April 2021)) in tQL, BL1 and BL2, respectively. As the magnetization parallel to k is vanishing, only the perpendicular component is shown, where values between −0.4 and 0.4 are mapped onto a colorbar from blue to red. The line width in these panels is proportional to the band projection sum of the respective atom group. The fifth and last panel is an image of the structure with the values of the reaction coordinate as labels.

The topological character of a material is determined by the $Z_{2}$ invariant which is defined for an insulating system in which conduction bands and valence bands are well separated. Systems with inversion symmetry make the calculation of the invariant quite simple as only the parity eigenvalues of each Kramers pair have to be multiplied together at the four (2D) or eight (3D) time reversal invariant momenta (TRIMs, $\Gamma_{i}=\frac{1}{2}\left(n_{1}b_{1}+n_{2}b_{2}\left[+n_{3}b_{3}\right]\right),n_{i}\in Z_{2}$) [28,29]. This procedure identifies ${\mathrm{Bi}}_{2}{\mathrm{Se}}_{3}$ with a 3D $Z_{2}$ insulator and a single Sb BL with a 2D trivial insulator. The double BL sheets are also topologically trivial as they are adiabatically connected to two copies of a single BL (green, yellow and red bands in Figure 4i–m). However, the $Z_{2}$ invariant is not defined for the present systems because the BS surface is metallic and does not provide a gap separating valence and conduction bands. The alternative approach for the determination of how the TSSs of the BS surface affect the bands of the Sb sheet is to identify the connection of the Kramers pairs at different TRIMs [29]. In detail, the Kramers pairs at the TRIM with inverted bands (the Γ point for ${\mathrm{Bi}}_{2}{\mathrm{Se}}_{3}$) are identified and its two branches are traced on their way to another TRIM. While in a trivial band structure, the two branches meet at the same Kramers pair at the end point after a facultative splitting, they connect two different Kramers pairs in a topological band structure. This is a direct consequence of the $Z_{2}$ invariant and the associated time reversal polarization pump [29].

**Figure 5:**
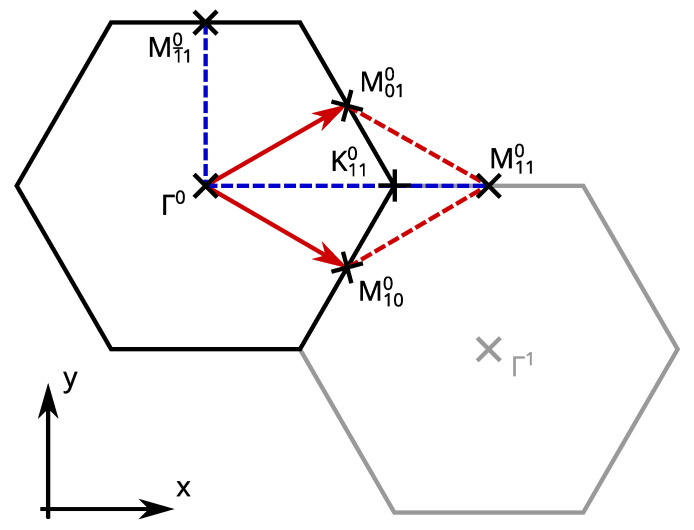
Brillouin zone and the four time reversal invariant momenta Γ^0^, $M_{10}^{0}$, $M_{10}^{0}$, and $M_{11}^{0}=M_{\bar{1}1}^{1}=M_{\bar{1}1}^{0}+G$. **Upper** indices denote the index of the Brillouin zone; **lower** indices are the half-integer coefficients of the reciprocal basis vectors.

As the BS surface is a 2D system, the Brillouin zone provides four TRIMs (Figure 5): the Γ point of the first Brillouin zone $\Gamma^{0}$, the two edge centers $M_{10}^{0}$ and $M_{01}^{0}$, and the point $M_{11}^{0}=\frac{1}{2}b_{1}+\frac{1}{2}b_{2}$, which is also an edge center and equivalent to $M_{\bar{1}1}^{0}=-\frac{1}{2}b_{1}+\frac{1}{2}b_{2}$. Because of the three-fold rotational cell symmetry, three M points at a time are equivalent. The time reversal symmetry connects two opposing M points, so the bands of one Γ-M path are sufficient for determining the Kramers pairs connection. For Figure 4, the $\Gamma^{0}$-$M_{\bar{1}1}^{0}$ path was chosen as it points into the *y*-direction, so the in-plane magnetization has an *x*-component only. In addition, the bands of the $\Gamma^{0}$-$M_{11}^{0}$ path are plotted, as it points into the *x*-direction (in-plane magnetization in *y*-direction) and contains the high-symmetry point $K_{11}^{0}$. This completes the picture of the band structure and makes the helicity of the spin expectation value comprehensible.

The bands of the 2BL-Sb@BS structure (Figure 4a) have three important Kramers pairs at the TRIM Γ^0^: The Dirac point D*, the Rashba-like crossing of the $R_{\mathrm{BS}}$ bands (labeled R) and the vertex of the $P_{\mathrm{Sb}}$ peak (labeled P). The lower branch originating in P connects to some state in the BS bulk bands on its way to the TRIM $M_{\bar{1}1}^{0}$. The upper branch goes first upwards, then bends down and seems to approach the lower branch coming from R. They merge, however, with the bulk BS valence bands near $M_{\bar{1}1}^{0}$ and do not form a Kramers pair. Instead they each form a Kramers pair with a state from the back surface as indicated by their weak surface localization (small line thickness). The lower branch of R connects to the valence bands after a short rise and comprises $R_{\mathrm{BS}}$ and the TSS from Figure 3. The upper branch rises more steeply and meanders to a Kramers pair in the conduction bands at $M_{\bar{1}1}^{0}$. Thus, R and its branches are topologically protected. The lower branch of D* tends to the Kramers pair connected with R, but it is disrupted by the bulk BS conduction bands and has, therefore, lost its topological protection.

When the first BL is lifted (Figure 4a–e), no phase transition in the sense of a Kramers pair connection interchange occurs. Thus, the bands of 1BL-Sb@BS and 2BL-Sb@BS can be transformed continuously into each other. The main transformation is a real-space migration of the TSS from the space between BL1 and BL2 (yellow-green) to the space between tQL and BL1 (blue-green). Simultaneously, the states of D* are transferred from BL2 (red) to BL1 (green). On the energy scale, D* drops, while P rises. Interestingly, the gap between the TSS and $R_{\mathrm{BS}}$ closes at $d_{2}=3.8$ Å (Figure 4d) so that D* seems to be connected to a Kramers pair in the bulk valence bands in this direction. However, the topological protection is not given as the intersecting bands have the same magnetic character and can, therefore, be continuously separated again. Figure 4e represents a 1BL-Sb@BS structure (with slightly reduced $d_{1}$) and an isolated, free-standing 1BL-Sb sheet.

Lifting BL1 (Figure 4e–i) involves a series of topological phase transitions. The first transformation is a further drop of D* in energy so that it dips into the bulk BS conduction bands. At $d_{1}=3.1$ Å (Figure 4f), D* seems to take the lower Kramers pair partner from R which would implicate topological protection. This is, however, not clear to tell because of the presence of the bulk BS conduction bands and $R_{\mathrm{BS}}$. At $d_{1}=3.6$ Å (Figure 4g), D* touches P and they change partners. Now the vertex of the upper peak (labeled $P_{1}$) has both its branches connected to the same Kramers pair at $M_{\bar{1}1}^{0}$ and is, therefore, topologically trivial. The vertex of the lower peak (labeled $P_{2}$) is connected to different Kramers pairs in the valence and conduction bands. In addition, a new Kramers pair (labeled D’) emerges from the bulk BS valence bands with its lower branch connected to somewhere in the valence bands. Its upper branch tends to the lower branch of $P_{2}$. This entanglement is lifted when BL1 moves further away from the surface and the gap in the lower Sb parabola closes. $P_{2}$ is then trivially connected and D’ becomes topologically protected. This case (Figure 4i) resembles the clean BS surface with its characteristic Dirac cone D’ at Γ and two free standing Sb bilayers, each with two trivially connected peaks $P_{1}$ and $P_{2}$. It should be noted that the situation in Figure 4i does not show a complete isolation of the three subsystems because BL1 still hybridizes with the BS surface bands. Further lifting, however, would initiate the merging between BL1 and BL2. From the following snapshots, one can derive that the separation between the BL and the BS surface completes at distances $d_{1}>6$ Å.

The next part of the path is the joining between the two Sb BL sheets to form a free-standing 2BL sheet (Figure 4i–m). Already at a distance of $d_{2}=5.0$ Å (Figure 4j), the two 1BL-Sb band structure copies begin to mix such that the bands cannot be assigned to a single BL anymore. After the merging, the top peak has transformed into an “M” (Figure 4m). As no gap closing occurs in the bands of the BL1-BL2 subsystem, the free-standing 2BL-Sb sheet has the same trivial topological class as the free-standing 1BL sheet. However, the band gap of the sheet reduces by a considerable amount from 1.3 eV (Figure 4i, fourth panel) to less than 0.1 eV, which agrees with Reference [22].

The last step is the readsorption of the double bilayer to the BS surface. At a distance of $d_{1}=4.8$ Å, the trivial Sb band hybridizes with the Dirac cone of the BS surface. After the partner change, the upper branch of D’ connects to the same Kramers pair as the lower branch of $P_{1}$. Because the upper branch of $P_{1}$ is now moving into the conduction bands, the top valence band; therefore, the whole Sb sheet, becomes topologized. However, like in the 1BL case, the branches of $P_{1}$ cross the BS bulk conduction bands, so the topological protection of the whole state is not given. Indeed, as the Sb sheet approaches the BS surface, a gap opens at the crossing between the TSS and the $R_{\mathrm{BS}}$ band first in the $\Gamma^{0}$-$M_{\bar{1}1}^{0}$ direction and then in the $\Gamma^{0}$-$M_{11}^{0}$ direction (Figure 4p). The Kramers pair which connects the valence bands to the conduction bands switches from $P_{1}$ to R. Furthermore, the second peak $P_{2}$ rises, and, when it touches D’, they both interchange partners (Figure 4p). This restores the original 2BL-Sb@BS band structure, thus closing the loop (Figure 4a).

An interesting feature during the closed readsorption cycle is the ordering of the Kramers pairs in the conduction bands at the M point. The index of the lowest Kramers pair (which is split due to back surface effects) in $M_{\bar{1}1}^{0}$ shifts by one after one cycle, as the conduction band of the Sb sheet pushes below. Thus, the development of the electronic band structure as a function of the interlayer distance yields valuable insight to understand the nature of the electronic states and the topologization process.

## 3. Discussion

### 3.1. Structural Details

Laterally shifting the Sb adlayer across the BS surface imposes a clear penalty on the Sb chemical potentials and on the stability of the structure. The main reason for this is the $p_{z}$ orbitals of the top Se atom which push the adlayer away from the BS surface and reduce the binding energy. Only when both Sb atoms fit into the space between the $p_{z}$ orbitals, the symmetry positions H3 and T4, the adlayer adsorbs close to the surface maximizing the binding energy. This compares well to the results in Reference [20].

In all investigated Sb@BS systems, inverting the adlayer has only a weak effect on the Sb chemical potential and, hence, on the structural stability. In the 1BL-Sb@BS case, the BL can interact only with the BS surface below it. Therefore, the distance $d_{1}$ is the main parameter controlling the binding energy. Because the natural stacking order with the lower Sb atom at the H3 site (above the BS surface hole) and the other one at T4 (not above the top Se atom) lets the BL come slightly closer to the BS surface than the one with swapped positions, the former maximizes the binding energy, while the latter is unfavourable by only 10 meV per Sb atom. This is consistent with Reference [18], who found that the natural and the inverted 1BL-Sb@BS structures are de facto degenerate.

In the 2BL-Sb@BS case, the weak influence of the BL inversion on the binding energies is quite surprising. As the inverted Sb bulk structure has $\mu_{\mathrm{Sb}}^{\mathrm{b-inv}}=40$ meV, a penalty of 20 meV per inversion would be expected. In particular, however, the twice inverted P1012 structure has a penalty of $\Delta \mu_{\mathrm{Sb}}=\left(45-33\right)/2\;\mathrm{meV}=6$ meV per inversion, only 25% of what was expected. The reason for the apparent paradox lies in the presence of the BS substrate, which requires a surface lattice constant of $a_{\mathrm{BS}}=4.13$ Å for lattice matched adsorption. A free-standing, single β-Sb sheet is smaller ($a_{\mathrm{Sb}}^{f1\mathrm{BL}}=4.05$ Å, −2.1%), so it has to be stretched to match the BS surface. On the other hand, the lattice constant of bulk β-Sb with natural stacking order is too large ($a_{\mathrm{Sb}}^{\mathrm{b-nat}}=4.31$ Å, +4.1%). A free-standing 2BL sheet of naturally stacked Sb lies in between with $a_{\mathrm{Sb}}^{f2\mathrm{BL-nat}}=4.16$ Å, +0.7%, so it matches the BS surface almost perfectly. When the Sb BLs are stacked in inverted order, the lateral dimensions grow by a smaller amount with $a_{\mathrm{Sb}}^{f2\mathrm{BL-inv}}=4.08$ Å, −1.2%, for the free-standing 2BL sheet and $a_{\mathrm{Sb}}^{\mathrm{b-inv}}=4.12$ Å, −0.4%, for thick layers (bulk limit).

The inverted and not-inverted versions of free-standing 2BL-Sb sheets do not only have similar lateral dimensions which match the BS surface almost perfectly. They also have similar chemical potentials ($\mu_{\mathrm{Sb}}^{f2\mathrm{BL-nat}}=178$ meV and $\mu_{\mathrm{Sb}}^{f2\mathrm{BL-inv}}=173$ meV). Consequently, the inversion of layers in the 2BL-Sb@BS structures has only a weak impact on the stability and the four investigated structures above are de facto degenerate.

When the Sb adsorbate becomes thicker, naturally stacked layers have to be squeezed in order to match the BS surface lattice constant $a_{\mathrm{BS}}$, which lifts the Sb chemical potential to $\mu_{\mathrm{Sb}}^{\mathrm{b-str}}=46$ meV in the bulk limit. On the other hand, inverted stacked layers already match the BS surface, so their Sb chemical potential is only a little above $\mu_{\mathrm{Sb}}^{\mathrm{b-inv}}=40$ meV. Hence, also for the adsorption of thicker layers, the insensitivity against layer inversion can be expected.

Concerning the experimental reality, we expect that the Sb adlayers form on the BS surface in a way that the two Sb atoms of the next bilayer occupy the H3 and the T4 site. Structures where one Sb atom sits above the top atom of the layer underneath are discouraged and not expected to form under real experimental conditions. Layer inversions, however, change the Sb chemical potential by less than 10 meV which is below the thermal energy $k_{B}T$. Hence, we expect that that the formation of local islands with different stacking order is possible.

### 3.2. Band Structures

The peel-off and readsorption process gives a detailed insight into how the BS surface affects the trivial Sb sheet. Below a distance of about 5 Å, the Sb sheet hybridizes with the BS Dirac cone and undergoes a topological phase transition. The Kramers pairs at different TRIMs change partners until the top Sb valence band connects to the conduction bands (“topologizes”). However, its Dirac point D* lies in the BS bulk conduction bands, which triggers another partner change at the crossing. As a result, D* loses its topological protection because both its branches go into the conduction bands. The topological property of the whole system is now preserved by the bottom edge of the BS conduction bands, where two bulk Kramers pairs merge to two Kramers pairs localized at each surface. One branch is still connected to the conduction bands, and the other one connects to the TSS which goes into the valence bands. As a consequence, the electron pockets are pulled apart so that they adopt a Rashba-like shape.

The hybridization of the Sb bands with the topological BS surface can partially be predicted by the conclusion of Reference [23]. However, the adsorption of a single Sb BL, in particular, involves a series of partner changes, i.e., at first, the lower Sb peak hybridizes with the TSS and then changes partners with the upper Sb peak. As the inter-layer distance controls this issue, an interesting approach to avoid the Dirac point going into the BS bulk states would be to lift the Sb adlayer, e.g., by ion intercalation.

The band structures of 1BL-Sb@BS and 2BL-Sb@BS are adiabatically connected, i.e., the adsorption of a second Sb BL on 1BL-Sb@BS does not involve any topological phase transitions in the Fermi energy region. The reason for this is the band alignment so that the bands of the 1BL-Sb@BS system lie in the band gap of the adsorbing BL [23]. The real-space distributions of the electronic bands just migrate continuously to the top Sb BL, which is consistent with Reference [24], and the band energies shift relatively to the bulk potential and to each other.

The dependence of the band shifts on the interlayer distance also explains why the band structures of the inverted adsorbates are similar to their non-inverted counterparts. Notably, lifting the BLs reproduces the same changes in the band structures as the layer inversions (compare Video S1, Structure 4, 20, and 54 with Figure 3b,d,f, respectively). This supports the conclusion that the main effect of layer inversion on the band structure is the change in the interlayer distances due to geometry. In particular, the gap opening in the TSS is driven by the interaction of the Sb adlayer with the BS surface in the sense that smaller interlayer distances lead to larger gaps and, therefore, reduce the total energy of the system. This explains why the natural stacking order, which minimizes the distances, is energetically favoured, even though by a small amount.

In the present case, DFT (and its VdW extension) describes the total-energy-related quantities (e.g., the forces and the equilibrium geometry) correctly, as they depend on the correct electronic occupation. However, the band structures given in this work are the DFT eigenvalues and cannot be translated directly to real excitation energies measured, e.g., in photoemission experiments. They can be corrected by the GW approach, which accounts for the correct self-energy of the quasiparticles. The impact of GW on bulk BS and the clean BS surface has already been investigated [30,31,32,33,34,35,36,37]. The main conclusion for the bulk system is that the bulk conduction bands shift everywhere except in the vicinity of the Γ point, where the band inversion leads to a lowering of the conduction bands and a rise of the valence bands. Consequently, unlike in trivial semiconductors, the band gap in bulk BS decreases after the application of GW [36]. The topological character, however, is still preserved, i.e., the BS substrate is still a 3D topological insulator [33,36]. In thin films of ${\mathrm{Bi}}_{2}{\mathrm{Se}}_{3}$, GW can change the topological character from the quantum-spin-Hall phase to the trivial phase because the shifting of the conduction bands away from the valence bands can reverse the band inversion [36]. Therefore, GW should retain the triviality of the thin Sb layers in this work and, thus, the existence of topological phase transitions during adsorption on BS. Furthermore, the band structures in this work are calculated with a very similar approach to that in Reference [24], where the key features of P01 and P0120 (Figure 3a,c) in this work reproduce well the experimental ARPES images of the system. For clean BS, the DFT band structure in Reference [24] compares well to that in References [35,36], where GW corrections shift the BS bands by ∼0.1 eV near the Γ point. Extrapolating this onto the Sb@BS systems here, we expect that, apart from potential band offsets caused by a changed Fermi level, the main conclusions of this work will not be affected by a GW correction. Nevertheless, this is an interesting point to be investigated in future work.

## 4. Materials and Methods

### 4.1. Computational Parameters

All DFT calculations were carried out with the Vienna ab initio simulation package (VASP) [38,39]. Bi, Se, and Sb were modeled by projector-augmented wave (PAW) potentials [40,41] with $5d^{10}6s^{2}6p^{3}$, $4s^{2}4p^{4}$, and $5s^{2}5p^{3}$ valency, respectively. The exchange-correlation energy was described by the generalized gradient approximation (GGA) as proposed by Perdew, Burke, and Ernzerhof (PBE) [42]. The kinetic-energy cutoff was set to 400 eV and the Brillouin zone of the hexagonal bulk/surface was sampled by a 12×12×3/12×12×1 Γ-centered grid [43], so that the total energies are converged within 1 meV/atom. Hellmann-Feynman forces [44] are used for the determination of the atomic equilibrium positions. For all calculations (relaxations, total energy, band structures), spin-orbit coupling (SOC) was included. The van der Waals (vdW) interaction was accounted for by correcting the DFT total energy with the DFT-D2 method [45].

### 4.2. Cell Optimization and Supercells

The hexagonal unit cells of bulk ${\mathrm{Bi}}_{2}{\mathrm{Se}}_{3}$ and Sb were optimized at constant volume, the equilibrium of which was taken from the minimum of the Murnaghan equation of state [46]. The free standing β-Sb and the inverted 2BL β-Sb sheet were optimized with the 2D version of Reference [46], the naturally stacked 2BL sheet with nested intervals up to an uncertainty of $0.02\;{Å}^{3}$.

Based on the optimized BS bulk cell, centrosymmetric supercells were build by stacking $2\;c_{\mathrm{bulk}}$ (≡6 QL) of substrate and $2\;c_{\mathrm{bulk}}$ of vacuum. The resulting slab provides two equivalent surfaces which are separated from each other by sufficiently thick bulk and vacuum so that the TSSs do not interfere with each other. In fact, the Dirac cone of the clean BS surface forms already at a slab thickness of 3QL. This is a known discrepancy of DFT-GGA calculations of BS [35]. The charge densities of the TSSs of both surfaces, however, are nonzero at the slab centre which indicates that the two surfaces still interfere. The slab thickness has to be increased to 6QL until the TSSs have vanishing charge densities at the slab centre. During structural relaxation, the inner 4 QLs were fixed, while the outer QLs and the adsorbed Sb BLs were free to relax in the *z* direction. The enforced bulk-like region accounts for the semiinfinite bulk substrate below the surface and accelerates the structural convergence.

## 5. Conclusions

In summary, we have investigated the morphological details of the ${\mathrm{Bi}}_{2}{\mathrm{Se}}_{3}$ surface with lattice-matched, adsorbed Sb sheets and elucidated their impact on the electronic band structure. The structures in which the Sb bilayers continue the stacking order of the underlying ${\mathrm{Bi}}_{2}{\mathrm{Se}}_{3}$ substrate have the largest binding energy. However, the penalty for inverting the Sb bilayers is below 10 meV per atom and, thus, small compared to $k_{B}T$. Our calculations suggest that inverted structures may also form under real experimental conditions.

Thin Sb layers undergo a topological phase transition when they adsorb on ${\mathrm{Bi}}_{2}{\mathrm{Se}}_{3}$. At an inter-layer distance below 5 Å, the Kramers pairs start a series of partner changes until the top Sb valence band transforms into new topological surface states which cross in D* at Γ. However, the Dirac cone is interrupted by the BS conduction bands with the consequence that D* is not topologically protected anymore. Instead, the bottom edge of the ${\mathrm{Bi}}_{2}{\mathrm{Se}}_{3}$ conduction bands inherits the connection to the valence bands, which enables the opening of a gap between D* and the topological surface states.

The peel-off and readsorption process gives access to the details about how the surface of a topological insulator reorganizes the bands of a trivial adlayer. Tracing the connection between Kramers pairs at different time reversal invariant momenta proves to be a quite effective way to follow the topological phase transition and might be the key for understanding the topologization of many other 2D materials.

## Figures and Tables

**Table 1:** Cell parameters for bulk ${\mathrm{Bi}}_{2}{\mathrm{Se}}_{3}$: the lateral lattice constant $a_{\mathrm{BS}}$ of the hexagonal cell, the cell height $c_{\mathrm{BS}}$, the cell volume *V*, the Wyckoff position parameters of the trigonal cell *u* and *v*, the inter-layer distance $d_{\mathrm{BS}}$, and the layer thickness $w_{\mathrm{BS}}$. The experimental reference is taken from [26]. The calculations include spin-orbit coupling and van der Waals interactions (DFT-D2) during relaxation. The deviation of the calculations from the experiment is given as percentage.

	$a_{\mathrm{BS}}$ [Å]	$c_{\mathrm{BS}}$ [Å]	*V* [Å^3^]	*u*	*v*	$d_{\mathrm{BS}}$ [Å]	$w_{\mathrm{BS}}$ [Å]
Experiment	4.143	28.636	425.67	0.4008	0.2117	2.579	6.966
Calculation	4.13	28.73	425.26	0.4000	0.2111	2.56	7.02
Deviation	0.22%	−0.34%	−0.55%	0.19%	0.26%	0.89%	−0.79%

**Table 2:** Calculated cell parameters for different bulk and layered β-Sb structures: the Sb chemical potential $\mu_{\mathrm{Sb}}$ (referred to that of bulk β-Sb), the lateral lattice constant $a_{\mathrm{Sb}}$ of the hexagonal cell, and the inter-layer distance $d_{\mathrm{Sb}}$ between the bilayers (BLs).

System	Abbreviation	$\mu_{\mathrm{Sb}}-\mu_{\mathrm{Sb}}^{\mathrm{b-nat}}$ [meV]	$a_{\mathrm{Sb}}$ [Å]	$d_{\mathrm{Sb}}$ [Å]
Bulk (natural stacking)	b-nat	0	4.31	2.16
Bulk (matched to BS)	b-str	46	4.13	2.16
Bulk (inverted stacking)	b-inv	40	4.12	2.84
Bulk (distorted stacking)	b-dis	47	4.06	2.94
Free-standing 1BL sheet	f1BL	276	4.05	—
Free-standing 2BL sheet (natural stacking)	f2BL-nat	178	4.16	2.43
Free-standing 2BL sheet (inverted stacking)	f2BL-inv	173	4.08	2.97

## Data Availability

Data shown in this paper is available from the authors on reasonable request.

## Supplementary Materials

The following are available at https://www.mdpi.com/1996-1944/14/7/1763/s1, Video S1: Complete set of band structure snap shots of the closed Sb adlayer peel-off and readsorption path. For the panelling, see Figure 4.
